# A Nonparametric Mean-Variance Smoothing Method to Assess *Arabidopsis* Cold Stress Transcriptional Regulator *CBF2* Overexpression Microarray Data

**DOI:** 10.1371/journal.pone.0019640

**Published:** 2011-05-17

**Authors:** Pingsha Hu, Tapabrata Maiti

**Affiliations:** 1 Department of Energy-Plant Research Laboratory, Michigan State University, East Lansing, Michigan, United States of America; 2 Department of Statistics and Probability, Michigan State University, East Lansing, Michigan, United States of America; Instituto de Biología Molecular y Celular de Plantas, Spain

## Abstract

Microarray is a powerful tool for genome-wide gene expression analysis. In microarray expression data, often mean and variance have certain relationships. We present a non-parametric mean-variance smoothing method (NPMVS) to analyze differentially expressed genes. In this method, a nonlinear smoothing curve is fitted to estimate the relationship between mean and variance. Inference is then made upon shrinkage estimation of posterior means assuming variances are known. Different methods have been applied to simulated datasets, in which a variety of mean and variance relationships were imposed. The simulation study showed that NPMVS outperformed the other two popular shrinkage estimation methods in some mean-variance relationships; and NPMVS was competitive with the two methods in other relationships. A real biological dataset, in which a cold stress transcription factor gene, *CBF2*, was overexpressed, has also been analyzed with the three methods. Gene ontology and cis-element analysis showed that NPMVS identified more cold and stress responsive genes than the other two methods did. The good performance of NPMVS is mainly due to its shrinkage estimation for both means and variances. In addition, NPMVS exploits a non-parametric regression between mean and variance, instead of assuming a specific parametric relationship between mean and variance. The source code written in R is available from the authors on request.

## Introduction

**Figure 1:**
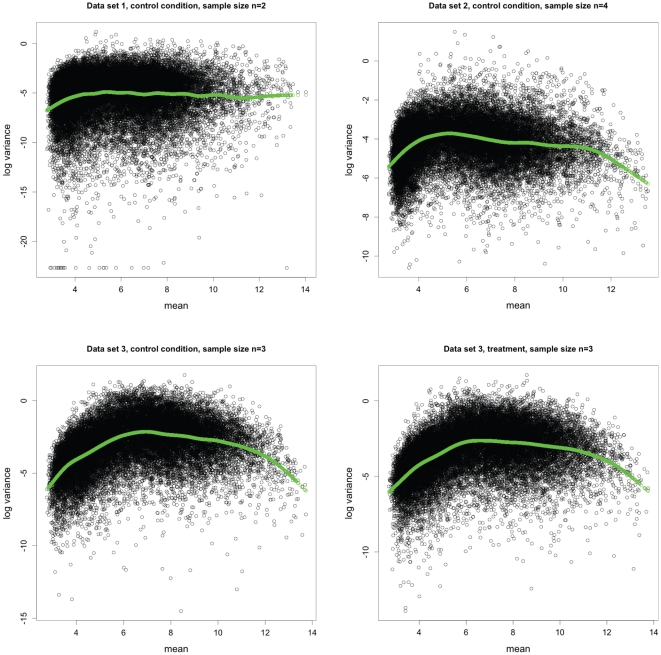
Relationship between sample mean and sample variance. Sample mean versus log sample variance plots of three different datasets from either control or treatment conditions. Smoothed variances using a non-paramteric method [6], [7] is displayed with green lines. Sample size n is indicated for each dataset. The data sets were normalized with RMA method.

Microarray has become a powerful tool for biological and medical science to monitor transcriptome changes under different treatments. However, because of high price of microarray experiments, replicates for each experiment are restricted in most cases. The feature of small replicates and large gene numbers, e.g., about 6,000 in yeast and 23,000 in Arabidopsis, in microarray data usually results in poor estimation of gene-specific variances. Several methods have been suggested for modification of gene specific variances or covariances to improve the estimation. For example, Efron et al. [1] suggested modifying the denominator of the 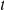-statistic to allow estimation less sensitive to gene-specific variances. Smyth [2] proposed smoothing gene-specific variances to a common value. Cui et al. [3] and Tong and Wang [4] developed shrinkage estimators for gene specific variances using Stein-type estimation under squared error loss function which were used to construct traditional 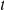
- type and 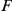
- type statistics. In all the above estimators, gene specific means were assumed to be independent of variances. It has been observed that means are related to variances in microarray experiments; usually genes with high expression level show high variances, while genes with low expression level display small variances (Figure 1).

Recently, Hu and Wright [5] suggested a linear model to estimate gene-specific variances based on means. However, the relationship between mean and variance is not always linear. Figure 1 shows real biological datasets from *Arabidopsis thaliana* in Gene Expression Omnibus database (GSE5566, GSE9955, and GSE5520 for dataset 1, 2, and 3, respectively) and clearly suggests a non-linear relationship between mean and variance. Here, we propose NPMVS (Non-Parametric Mean Variance Smoothing), a method to estimate the mean and variance relationship, which is more general and can capture a wider range of non-linear relationships that exist in microarray experiments (Figure 1). We explore the mean-variance relationship by fitting a nonlinear curve using penalized splines [6], [7]. In addition, inference is made upon shrinkage estimation of posterior means from Empirical Bayesian perspectives in our model, instead of 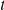-statistic, which was used by Hu and Wright to test differential expression. Therefore, our approach has shrinkage estimation of both means and variances. First variances are smoothed using means, then means are smoothed assuming the variances are known. The simulation results showed that, under different mean-variance relationships, our method outperformed or was competitive with the other two popular shrinkage estimation methods, limma [2] and Gottardo et al. [8] generalized Bayesian statistic model 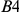, which assumes separate means and variances under different treatments for a given dataset. We also applied the three methods to a real biological dataset [9] to identify genes in cold stress regulatory pathways. With NPMVS, we detected more genes in the pathways and uniquely identified transcriptional changes in cell wall metabolism-related components under overexpression of a key transcription factor for freezing tolerance, *CBF2*.

## Results and Discussion

### Analysis of simulated data

**Figure 2:**
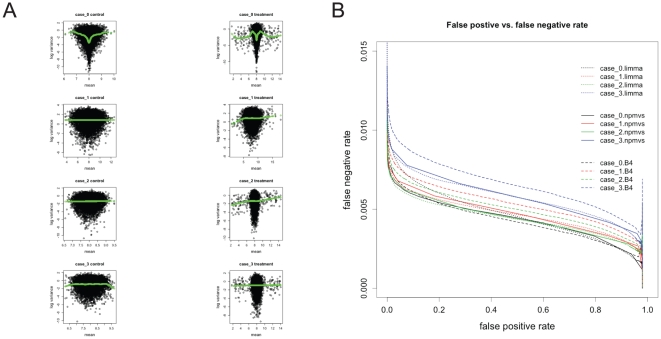
Simulation results from four mean-variance relationships. (A) The plots display mean versus log variance relationship in the four simulated data from case 0 to case 4. Simulated control data are presented on the left, and differentially expressed data are on the right. Smoothed variances using a non-paramteric method is displayed with green lines. (B) The plot displays false negative versus false positive rate for identifying DE genes in the simulated data using different methods. The false positive and false negative rate are the average rate from 100 simulated datasets. They were estimated over a range of cut-off values for each method. Dashed line, solid line, and dotted line represent Gottardo et al. [8] Bayseian (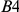) method, NPMVS and limma, respectively. Four mean and variance relationships, case 0, case 1, case 2, and case 3 are represented by black, red, green and blue colours, respectively.

To evaluate the performance of NPMVS, we compared it to the other two established methods, limma [2] (http://bioconductor.org/packages/2.5/bioc/html/limma.html), a linear model approach with variance shrinkage, and the 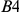 statistic from a Bayesian method [8]. We compared the performance of the three methods using simulated datasets. For each simulated dataset, a pair of data for control and treatment was generated with 10,000 genes. For the control data, we assumed that all genes expression level has a normal distribution with mean at 8 and standard deviation of 1. For the treatment data, 200 (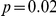), 500 (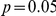), and 1000 (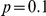) genes were assigned as differentially expressed (DE) ones in different data designs. The up- and down-regulated DE genes were created with uniform distributions with different mean ranges. For up-regulated genes, 25% out of the total DE ones were assigned with mean from 8.1 to 11, another 25% from 11.1 to 14; For down-reulated genes, 25% were assigned with mean from 5 to 7.9 and another 25% from 2 to 4.9. Samples for each genes were simulated as independent normal observations with four mean (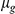) and variance (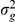) relationships (Figure 2A) (see methods for details). We simulated datasets with 3 replicates. For each dataset design, different DE gene percentage and mean-variance relationship were imposed. One hundred simulated datasets were generated per design.

A plot of type I and type II error curve was used to compare the performance of the three methods (Figure 2B). For datasets with different percentage of DE genes, we had similar results. Here, we showed a representative result when the DE gene rate 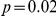. Figure 2B shows the performance of the three methods in different mean-variance relationships. NPMVS and limma are competitive in the three mean-variance scenarios except case 0, in which limma displayed higher type I and II error rate than the other two methods. Compared with 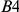 statistic, NPMVS had better performance. In three cases (case 1 to 3), NPMVS outperformed the 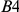 statistic. In case 0, where non-linear relationship was displayed for variance and mean, NPMVS has better performance than 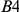 given a false positive rate less than 40% (Figure 2B).

### Analysis of *CBF2* overexpression line data

Higher plants have complex regulatory mechanisms to temperature changes. Cold acclimation is a process by which plants increase their freezing tolerance in response to low, non-freezing temperatures. Previous studies have demonstrated that in Arabidopsis cold acclimation rapidly induces the expression of *CBF* genes, key transcription factors in response to low temperature. CBFs can increase freezing tolerance through activating downstream target genes (the CBF regulons) by binding to the target genes promoter region. To gain a better understanding of the CBF regulatory network, gene expression profiles were generated between *CBF2* overexpression lines (*CBF2_OX*), which constitutively overexpresses *CBF2* and can tolerance freezing without prior cold acclimation, and wild type (wt) [9], [10]. The microarray data was analyzed by limma, 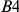 and NPMVS methods, respectively.

**Figure 3:**
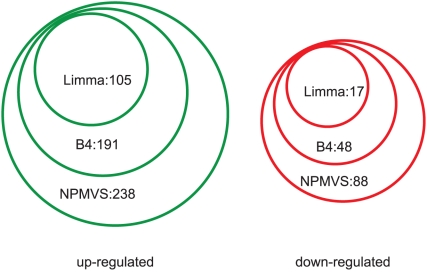
Identification of *CBF2_OX* differentially expressed genes. Up- and down-regulated genes greater than 2 fold changes are uncovered by three different methods with cut-off 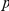 (adjusted by Benjamini & Hochberg method) value less than 0.01 for limma, and a cut-off posterior probability greater than 0.99 for 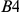 and NPMVS, respectively.

We retrieved DE genes with an adjusted cut-off 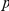 value less than 0.01 from limma, and a cut-off posterior probability greater than 0.99 from both 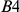 and NPMVS. The DE genes were further filtered with gene's average log 2 fold change greater than 1 or less than -1. As a result, NPMVS discovered more DE genes in both up- and down-regulated gene sets than limma and 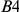 (Figure 3) did. Limma identified 105 up-regulated genes, while 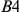 and NPMVS identified 191 and 238 up-regulated genes, respectively. In the down-regulated genes, limma only identified 17 genes, while 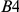 and NPMVS retrieved 48 and 88 genes, respectively. In addition, all genes identified by NPMVS were also found in the gene set identified by 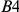 and limma. Some genes discovered by NPMVS but not limma, like transcription factor *RAV1*, sugar related genes and cell wall synthesis genes, have been identified as CBF and cold responsive genes previously [9], [11].

To evaluate the DE genes identified by the three methods, we accessed DE gene functions by gene ontology and cis-regulatory elements analysis to see if they are related to CBF and cold responsive pathway. Gene ontology enrichment analysis was performed on the three DE gene sets produced by the three methods (Table 1). Genes response to stress are enriched in all three up-regulated gene sets discovered by the different methods. The above result is consistent with the function of CBFs, which activates cold responsive genes as well as other abiotic stress responsive genes [12]. The gene set from NPMVS showed the most significant enrichment with the smallest 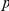 value compared to the gene sets identified by the other two methods (Table 1). Some important CBF2 target genes, such as *RAV1*, were missed when using limma but identified by NPMVS. Other genes like *RCI2A*(Rare Cold Inducible 2A), which is induced under various stress conditions [13], was also uniquely discovered by NPMVS. In the down-regulated gene set, limma did not find any enriched GO terms. From NPMVS and 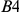, the GO term respond to stress was also enriched in the down-regulated gene sets.

**Table 1:** Gene ontology enrichment analysis for *CBF2_OX* up- and down-regulated genes.

	limma	*B*4	NPMVS
Enriched GO in up-regulated genes	response to stress (8.39E-09)	response to stress (2.82E-10)	response to stress (2.83E-12)
Enriched GO in down-regulated genes	N.A.	response to stress (0.034)	response to stress (0.00016)
	N.A.	cell wall (0.034)	cell wall (8.91E-06)

*P* values, which are indicated in parentheses, were adjusted by Benjamini & Hochberg method.

Enrichment of cell wall components has been uniquely discovered by NPMVS in the down-regulated genes. Most of these genes are involved in cell wall metabolism (Table S1). This result is in agreement with the previous report that cell wall related genes were down-regulated in the later time points (8 hour to 168 hour) under cold stress [11]. It has also been reported that cold acclimation resulted in increase of cell wall weight and a change in cell wall composition [14]. All above suggest CBF mediated cold responsive pathway is involved in the cell wall re-organization.

We also investigated enrichment of cis-regulatory elements in the up- and down-regulated genes identified from the three methods (Table 2). A cis-regulatory element usually is a 4-12 word nucleotide motif in a gene promoter region. Transcription factors activate/repress expression of their regulons by binding to cis-regulatory elements in the promoter of their target genes. The transcription factor CBF2 binds to a conserved cis-regulatory element, the CRT/DRE, which contains a CCGAC core motif, presented in CBF target gene promoter regions, and thus activates transcription of the CBF regulons. We expected that the CRT/DRE cis-element would be enriched in the up- and/or down-regulated gene sets, assuming that the direct CBF target genes were in the discovered gene sets. As a result, the most highly enriched cis-element is the CRT/DRE CCGAC core motif, which has been discovered in all up-regulated gene sets from the three methods. NPMVS identified 24 and 59 more genes containing CCGAC than 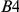 and limma did, respectively (Table 2). There are another two motifs identified in all three up-regulated gene sets with a cut-off 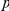 value less 0.0001 (Table 2). The NPMVS gene set also showed the most significant 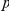 value of the second motif, ACGTG, which is an ABRE-like element that has been found in the promoter regions of cold, high-salinity and drought stress regulated genes [12]. Two additional enriched motifs have been identified by NPMVS and 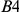 but not limma. Three motifs have been uniquely discovered by NPMVS in the up-regulated dataset. Two out of three motifs, CCACG and CACGTG, which contain the BOXII and CACGTGMOTIF core elements, respectively, are related to light response [15], [16]. The two motifs have not been reported in the previous CBF regulon motif studies [9]. No significant cis-acting elements have been found in all three down-regulated gene sets. The gene ontology and cis-regulatory element analysis indicated that NPMVS identified more stress responsive genes than limma and 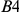 did.

**Table 2:** Enriched cis-regulatory elements in *CBF2_OX* up-regulated genes.

	Limma		*B*4		NPMVS		PLACE[20] Annotation
word	*p* value	counts	*p* value	counts	*p* value	counts	
CCGAC	7.45E-35	78	1.06E-43	113	1.36E-43	137	DRE
ACGTG	6.02E-06	69	2.48E-09	104	1.42E-11	133	ABRE
ATGTCG	9.30E-26	47	2.04E-33	65	6.64E-31	77	N.A.
CCACG			4.01E-06	73	1.09E-05	89	BOXIIPCCHS
CGGCA			9.04E-06	59	2.79E-06	71	N.A.
ACACG					9.95E-07	133	GADOWNAT
CACGTG					6.68E-05	55	CACGTMOTIF
CGTGTC					1.44E-05	51	N.A.

### Conclusions

In this paper we compared the three shrinkage estimation methods, limma, 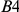, and NPMVS. Limma and 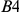 only have shrinkage estimation on variances, while our method NPMVS has shrinkage estimation on both means and variances. The simulation study showed that NPMSV performed better than limma in case 0, and the two methods were competitive in other mean-variance relationships. NPMSV outperformed 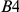 in case 1, 2, 3 and a competitor in case 0. The real microarray data from an overexpression line *CBF2*, which is a major regulator in cold and abiotic stress responsive pathways, was explored by the three methods. NPMVS identified more genes than both limma and 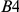 did. In addition, the gene set discovered by NPMVS included all genes identified by the other two methods. The gene ontology analysis showed that genes additionally identified by NPMVS are also related to stress response, which is consistent with previous findings for CBF2 targeted genes, implying that the NPMVS method makes a considerable improvement for gene detection. In agreement with gene ontology analysis, search of cis-acting elements in the up- and down-regulated gene sets showed that NPMVS identified more genes containing the core CBF response element, CCACG. NPMVS uniquely discovered genes involved in cell wall re-organization, which is consistent with previous cold stress microarray data [11]. Cis-acting elements, Box II and CACGTGMOTIF, which are light responsive components, were also uniquely discovered by NPMVS.

The good performance of NPMVS is mainly due to its shrinkage estimation for both means and variances. Our model used “smoothed” estimation of variances, which combines information from other genes. In addition, our method exploits mean and variance relationship, which is generally not considered in standard procedure. There is no specific type of relationship assumed for mean and variance; instead a nonparametric regression has been used. All above features contribute to the robustness of NPMVS. However, we should be aware that our NPMVS is based on the assumption that there is a relationship between mean and variance. Application of NPMVS will not be justified well if the assumption does not hold, namely, means are independent of variances. Mean and variance relationship should be investigated before the application of NPMVS.

## Methods

For shrinkage estimation of both means and variances, our objective first is to obtain smooth estimation of gene specific variances and then to use estimated variances in a hierarchical model assuming it is known. Therefore, our approach has two steps. First, variances are smoothed using means. Second, means were smoothed by a hierarchical model assuming their variances (improved estimated variances) are known. Here, we first present a general hierarchical model and how to make inferences about DE genes based on the Bayes rule. Then we propose non-parametric estimation of variances and a new hierarchical model, which assumes that smoothed variances are known and takes more general form of a prior. Finally, we present a multiple sample case hierarchical model with smoothed variances.

### Hierarchical model for one sample case

Let 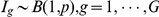, be the Bernoulli random variable indicating whether the gene 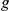 is differentially expressed 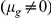, i.e., Prob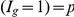, where
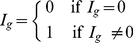
(1)and 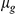 denotes the mean expression level for the gene 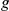. For each gene 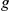 we are interested in knowing if the gene is differentially expressed given the data.
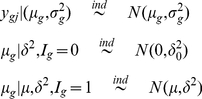
(2)


Then one can make inference on the basis of posterior probability
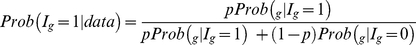
(3)where 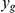 is the vector of measurement for gene 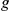. It is easy to see that the posterior mean of 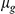 is 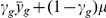, where 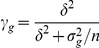. This type of hierarchical model was considered by Baldi *et al.* [17], Lonnstedt *et al.* [18], [19] and Gottardo *et al.* [8]. Moreover, in some of the above papers, 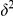 was taken in the form of 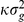 in which case the posterior mean does not even depend on gene specific variances and the shrinkage factors (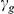) are constants. Although the estimators preserve the shrinkage, the assumption 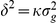 is hard to justify. In the case of 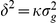, the posterior mean has a closed form expression. For example, the structure of the 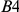 estimator is 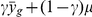, the shrinkage factor 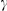 is constant over all genes. The only advantage of this is that the Bayesian computations get easier.

### Proposed Hierarchical Model and Smoothing Variances

We examined Arabidopsis Affymetrix microarray data and plotted the log variances with their mean (Figure 1). The plot immediately suggested no linear relationship is appropriate. Thus we fitted a nonlinear curve using penalized spline [6], [7]. Clearly, the spline did a very good fitting. Thus our objective is to first obtain the smooth estimate of the gene specific variances and then use them into a hierarchical model assuming they are known.

Smoothing variances:

We assume that the gene expressions 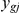 for 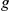
th gene and 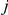
th replicate are normally distributed with mean 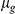 and variance 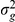; 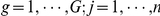, and define 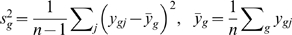, where 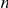 is the sample size in the given data. We assume the following two level model
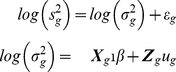
(4)where the 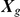 ans 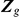 are constructed from sample means and their quantiles [7]. It is easy to obtain the best linear unbiased predictor of 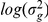 as 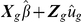. Note that all 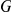 genes are being used in this smoothing process. Let 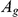 be the estimated values. To estimate the probability of differential expression, we modified (2) in the hierarchical model (1)-(3) as described below.
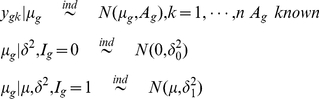
(5)


Lonnstedt and Speed [18], Gottardo *et al.* [8] and Lonnstedt and Britton [19] took 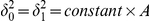. The above structure facilitates the posterior calculations in a closed form. However, we do not see much of reasoning that the between variance would be a constant multiple of the within variance. Therefore, our model is more general.

Identifying the posterior distribution of 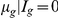 as 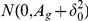 and 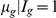 as 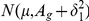, one can find
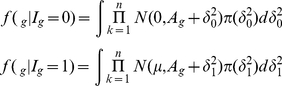
(6)where 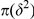 is the prior distribution of 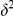. Previous works used Inverse gamma as a natural choice and the hyperparameter values were supplied. Note that again, unless 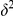 is a multiple of 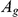, closed form expression does not exist even with IG prior distribution. Without any other prior information, we propose the uniform prior 
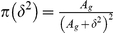
. In case of 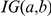 prior distribution, the above two conditional distribution takes the form
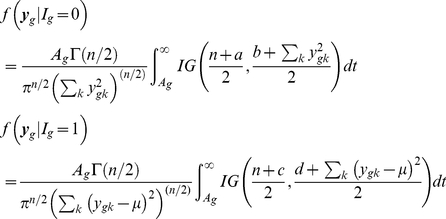
(7)


Choose 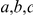 and 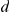 arbitrary positive number, say 0.5 or 0.01. They only involve evaluating the cumulative distribution function of inverse gamma distribution. Instead of using any prior distribution about 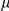, we shall use some pre-assigned quantity. The natural choice is to use grand mean expression value over all the genes.

If we use the uniform prior 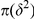, then the conditional distributions take the form
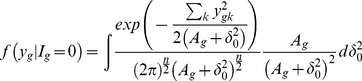
(8)

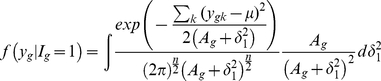
(9)


This is what we have used in our study.

### Multiple Sample Case



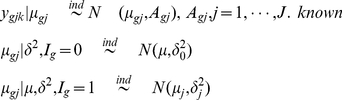
(10)Using the similar prior distribution for variance parameters 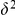 used in (7), we can easily shown that the distributions are
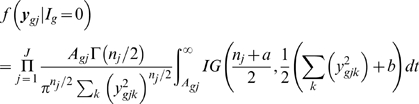
(11)

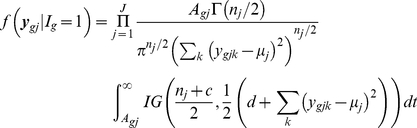
(12)


If we use the uniform prior 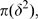 then the above conditional distributions are
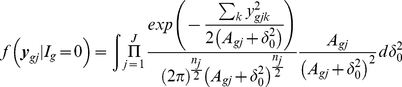
(13)

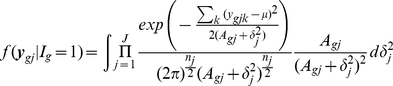
(14)


Note that *apriori* the 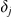
's are assumed to be independent. There is a number of possible modifications can be easily done. For example, one might assume same variance for all the conditions for non regular gene means or different variances for regular gene means.

We evaluated the one dimensional integral in (13) and (14) using 20 point Gauss-Hermite procedure.

### Method Evaluation

To evaluate the performance of NPMVS, we compared it to other two established methods, limma [2] (http://bioconductor.org/packages/2.5/bioc/html/limma.html) and Gottardo et al. [8] Bayesian method. We compared the performance of the three methods using simulated datasets and a real biological dataset, in which an overexpression line *CBF2_OX* was compared with wild type control.

#### Simulated Data

We applied different mean-variance relationships for the simulated data. First, expression means were generated from normal distribution for non-differentially expressed genes, 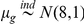; For DE genes, 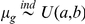, where 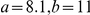 or 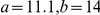 for up-regulated and highly up-regulated genes, respectively, and 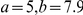 or 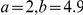 for down-regulated and deeply down-regulated genes, respectively. Secondly, variance 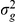 was generated, 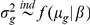. The plots of four mean-variance relationships are displayed in Figure 2A. Last, expression data 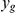 was generated from normal with mean and variance produced in the first two steps, 
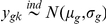
, where k = 3 is for three replicates. The most non-linear relationship is symbolized as case 0. In the simulation data, the choice of parameters 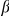 was based on the variance range observed from real datasets. The variance range produced in the simulation data was closed to the one surveyed from four real datasets (GSE9955, GSE5520, GSE5536, and GSE5727). Note that, since we used a nonparametric method, there is no need to estimate the beta parameters in real data application.

#### Real Biological Data

The CBF2 data (GEO series number: GSE5566) includes two genotypes, two independent *CBF2* overexpression lines and its corresponding *Arabidopsis thaliana* wild type, and two samples for each genotype. The microarray platform is Affymetrix ATH1 GeneChip. The raw CEL files were normalized by RMA. Fisher's exact test for one-tail (over-presented) was applied to the Gene Ontology enrichment analysis. The 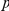 value for over-presented GO terms 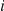 is 
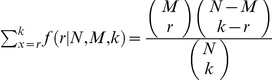
, where 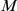 is the total DE gene number; 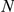 is the total gene number in the genome; 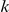 is the gene number in GO term 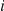; and 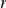 is the number of genes which belong to DE gene list in GO term 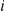. Benjamini and Hochberg false discovery rate correction was used for adjusting 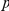 values. Five hundred base pair promoter region sequence for each gene was used for cis-regulatory element analysis via a *de novo* motif searching tool ELEMENT (http://element.cgrb.oregonstate.edu/). An enumerative method in ELEMENT was used for counting 4-8 mer DNA words. By comparing a word frequency for a given gene set to samples from the whole *Arabidopsis* genome sequence (a bootstrap procedure), a corresponding 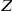 score and 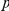 value (adjusted by Benjamin and Hochberg FDR method) were calculated in ELEMENT to estimate if the word is over-presented in the given gene set.

## Supporting Information

Table S1 (CSV)
